# Where tulips and crocuses are popular food snacks: Kurdish traditional foraging reveals traces of mobile pastoralism in Southern Iraqi Kurdistan

**DOI:** 10.1186/s13002-019-0341-0

**Published:** 2019-11-27

**Authors:** Andrea Pieroni, Hawre Zahir, Hawraz Ibrahim M. Amin, Renata Sõukand

**Affiliations:** 1 University of Gastronomic Sciences, Piazza Vittorio Emanuele 9, I-12042 Bra/Pollenzo, Italy; 2 Department of Biology, University of Raparin, Ranya, Kurdistan Region Iraq; 3 Department of Pharmaceutical Sciences, University of Eastern Piedmont, Largo Donegani 2, I-28100 Novara, Italy; 4 Department of Chemistry, College of Science, Salahaddin University, Erbil, Kurdistan Region Iraq; 5 Department of Environmental Sciences, Informatics, and Statistics, Ca’ Foscari University of Venice, Venice, Italy

**Keywords:** Wild food plants, Foraging, Ethnobotany, Kurds, Kakais, Human ecology

## Abstract

**Background:**

Iraqi Kurdistan is a special hotspot for bio-cultural diversity and for investigating patterns of traditional wild food plant foraging, considering that this area was the home of the first Neolithic communities and has been, over millennia, a crossroad of different civilizations and cultures. The aim of this ethnobotanical field study was to cross-culturally compare the wild food plants traditionally gathered by Kurdish Muslims and those gathered by the ancient Kurdish Kakai (Yarsan) religious group and to possibly better understand the human ecology behind these practices.

**Methods:**

Twelve villages were visited and 123 study participants (55 Kakai and 68 Muslim Kurds) were interviewed on the specific topic of the wild food plants they currently gather and consume.

**Results:**

The culinary use of 54 folk wild plant taxa (corresponding to 65 botanical taxa) and two folk wild mushroom taxa were documented. While Kakais and Muslims do share a majority of the quoted food plants and also their uses, among the plant ingredients exclusively and commonly quoted by Muslims non-weedy plants are slightly preponderant. Moreover, more than half of the overall recorded wild food plants are used raw as snacks, i.e. plant parts are consumed on the spot after their gathering and only sometimes do they enter into the domestic arena. Among them, it is worth mentioning the consumption of raw wild crocus corms, also still common in Turkish Kurdistan and that of wild tulip bulbs, which was documented to be popular until the beginning of the twentieth century in the Middle East. Comparison with other ethnobotanical field studies recently conducted among surrounding populations has shown that Kurds tend to gather and consume the largest number of non-weedy wild vegetables.

**Conclusion:**

The collected data indicate robust traces of nomadic pastoralism in Kurdish traditional foraging. This finding confirms that studies on wild food plant gathering in the Fertile Crescent and Turco-Arabic-Iranic regions of the Middle East are crucial for understanding the possible evolution of wild food plant gathering through history within the post-Neolithic continuum between pastoralism and horticulturalism.

## Introduction

Is Kurdistan truly an important hotspot for traditional gathering of wild vegetables in the Middle East? Are foraging patterns of different ethno-religious groups living in Kurdistan and surrounding areas similar, and if so, why? What are the possible origins of Kurdish foraging?

The current field study tries to address these questions by analysing the wild food ethnobotany of a broad area of Southern Iraqi Kurdistan ranging from the biblical Nineveh Plains in the west to the semi-desert area of the Garmian Region of Kalar to the east, i.e. a bordering plain area where the Kurdish population ends at the Arabic realm.

The region that we nowadays call Kurdistan, which is divided among the modern nation states of Syria, Turkey, Iraq, and Iran, and its surrounding territories are a very essential area for understanding the human ecology of food and the evolution of human nutrition patterns, as this area was home to a remarkable portion of the Neolithic Revolution. Some crucial archaeological sites in this northern and eastern part of the Fertile Crescent (i.e. Çayönü Tepesi, Göbleki Tepe, Jarmo, Mureybet, Tell Abu Hureyra, Tell Sabi Abyad; dating to 7500–10,500 BC) have traced the transition from hunting and gathering to the first sedentary settlements, as well as the domestication of cereals, pulses, and various mammals [1–4].

Moreover, in the very recent ethnobotanical literature, Kurdistan seems to emerge as a remarkable *bio-cultural food refugium* [5] for wild food plants and foraging customs, if compared with other Mediterranean and Near Eastern areas, especially in terms of the number of botanical taxa still currently utilized [6–13]. In addition, the Kurdish region is located at the crossroad of four important cultural areas (Turkish, Arabic, Persian, Caucasian) and still hosts diverse ethnic, linguistic, and religious minority groups who have peacefully lived together for centuries. In particular, we recently found that in Northern Iraq, affiliation to different religious communities, which possibly had an effect for centuries on kinship relations and then on the vertical transmission of Local/Traditional Environmental Knowledge and Practice (TEK) related to food within the household, has shaped different foraging patterns [14]. The differences we observed were especially remarkable between Christian Assyrians, whose wild food plants are mainly represented by synanthropic weeds, and Muslim Kurds, who favour wild plants growing in the mountains, and we postulated that this disparity could be related to the ethnogenesis of the two groups: post-Neolithic horticulturalists and nomadic pastoralists, respectively.

In the present study, which we conducted at the most southern edge of the Kurdish Autonomous Region in Iraq, we considered two diverse ethno-religious Kurdish groups: the Muslim and the Kakai (also named Yarsani) communities. The term *kurd*—possibly derived from the Middle Persian *kwrt*, meaning nomad or tent-dweller [14]—emerged in the sixteenth century to describe a few heterogeneous nomadic shepherding tribes living in the Central Persian Plateau, and their origins are probably to be found in different pre-existing civilizations, among them that of the Medes, which possibly also gave rise to the Baluchi people [15]. Kurds were mainly Islamicised by the Turks between the twelfth and the fifteenth centuries [16] while Yarsanism is instead a monotheistic faith which specifically emerged from Shia Islam in Western Iran in the fourteenth century [16].

A previous study that we conducted in Hawraman, SE Iraqi Kurdistan, showed that the wild food ethnobotanies of Yarsani (Kakai) and Muslim Kurds entirely overlap [17]. Despite the very small sample of Yarsani informants that could be considered, we interpreted this finding as the result of a rapid acculturation process that Yarsanis living in that area had to go through during the past two decades, when they were heavily threatened by the spread of radical Jihadism. As a consequence, Hawramani Yarsanis were forced to abandon their home village of Hawar and move to main Kurdish towns, where they lived together with their Muslim counterparts, returning to the village only during the summer months or at weekends [17].

In this study, we wanted to further verify this finding by using a broader sample which included Yarsanis still living in their original villages in the Nineveh and Garmian plains.

The specific research objectives of this study were therefore:

- To record the local names and specific traditional culinary uses of local wild food plants among Kurds living in different locations of Sothern Iraqi Kurdistan;
- To compare the data collected among the two considered religious/ethnic communities (Kakai and Muslim Kurds); and
- To compare the data with all the available Middle Eastern and Mediterranean wild food ethnobotanical literature, in order to possibly trace commonalities and differences, which could be linked to historical and/or human ecological dynamics.

## Methods

### Research area and field study

**Fig. 1:**
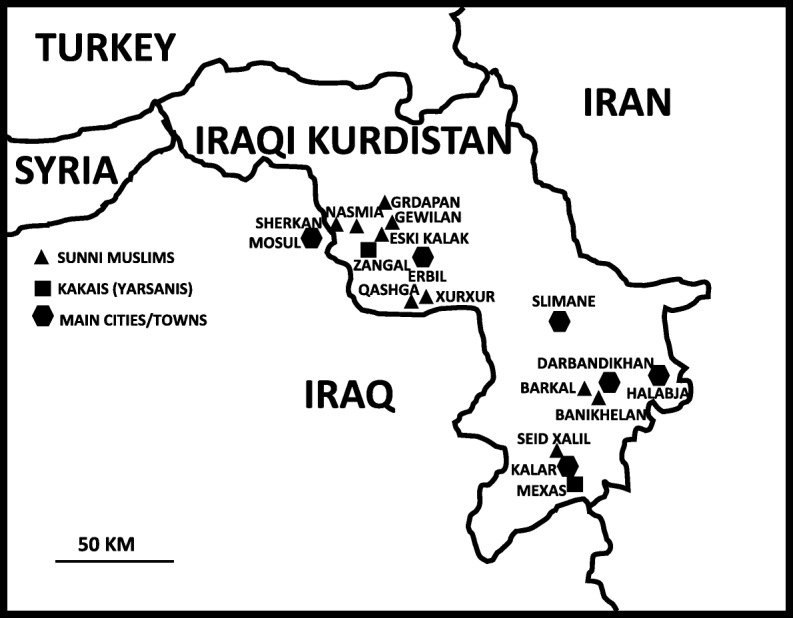
Map of the study area and villages

**Fig. 2:**
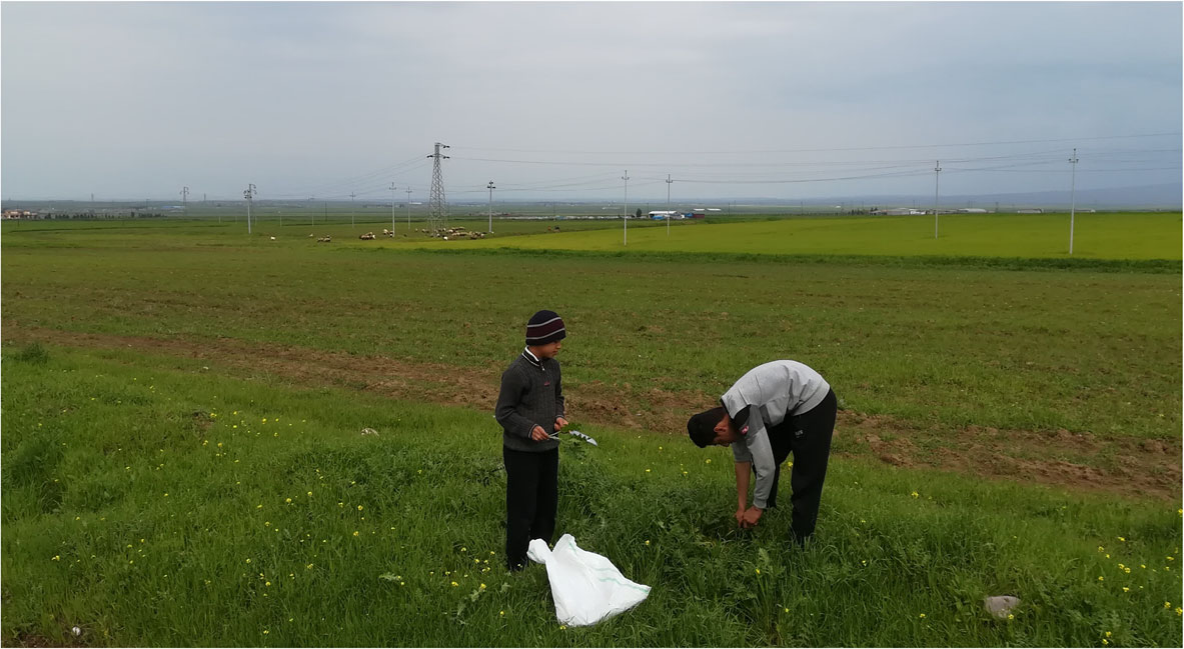
Two young foragers in the Nineveh Plain

**Fig. 3:**
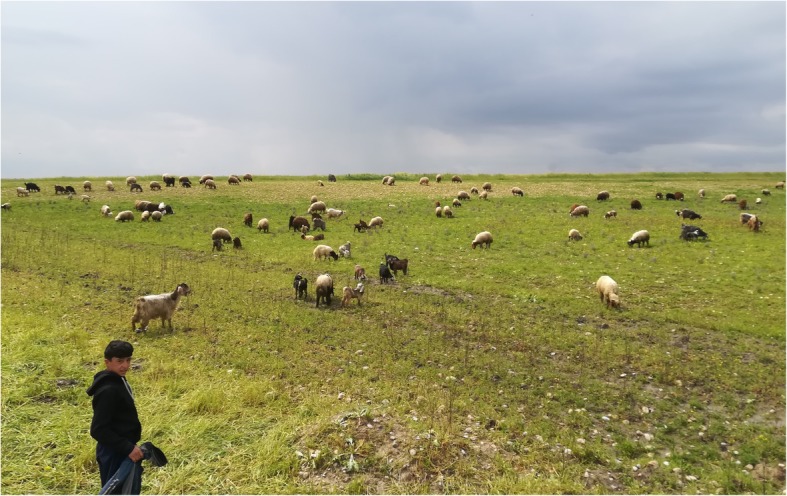
Shepherding in the Garmian region

The field study was conducted in Southern Kurdistan (within the border of Iraq) in the spring of 2019, during which time we visited 12 villages (Fig. 1) inhabited by Kakai and Muslim Kurds, with a population ranging between 100 and 5000 inhabitants. The villages are located mainly in plain areas along the plateaus cut by the lower Great Zab, Little Zab, and Sirwan rivers, which are all tributaries of the Tigris. The lower Great Zab River cuts the Biblical Nineveh Plain (Fig. 2), while the Sirwan River runs through the Garmian region (Fig. 3). The two religious communities we considered are strictly endogamic, and intermarriages are still not allowed, nor commonplace; both Muslim and Kakai Kurds speak Sorani Kurdish, the latter group often mixing this with their original Gorani (also called Gurani) language. Gorani is considered by linguists to be part of the Zaza-Gorani language which does not fall under the Kurdish language group, although it still belongs to the NW branch of Iranian languages [18]. Nevertheless, all Gorani speakers as well as Kakais consider themselves Kurds and they speak proper Kurdish too.

The vegetation of our study area belongs to the Armeno-Iranian Province of the Irano-Turanian Region [19] and, according to the Köppen-Geiger classification system [20], presents a hot semi-arid climate (BSh), with extreme temperatures in the summer that in the town of Kalar may easily reach 50 °C.

The villages we considered rely upon small-scale horticultural and especially pastoralist subsistence economies, with some of the younger and middle-aged population working in the public sector in the main cities.

Fifty-five Kakai Kurds (28 men and 27 women) and 68 Muslim Kurds (40 men and 28 women) aged between 31 and 79 years were interviewed. Respondents were selected among the middle-aged and elderly villagers who have a strong link to traditional agro-pastoral activities and still actively practise the custom of gathering wild plants. The focus of the interviews, which were conducted in Sorani Kurdish, was the local names, modalities of gathering, and detailed culinary uses of the *currently collected* wild food plants. The data were acquired also via free-listing exercises, group walks through the natural landscape, and participant observation.

Prior informed consent was verbally obtained before each interview, and the Code of Ethics of the International Society of Ethnobiology [21] was followed.

Villages of the Nineveh Plain are still heavily affected by the consequences of the occupation of Mosul and the surrounding territory by the terrorist groups of the former Islamic State of Iraq and Syria (ISIS, 2014–2017), with several internal refugees still living in camps, after the occupiers were expelled from Mosul city and its contiguous areas. Kurdish Peshmerga (military forces of the autonomous Kurdistan Region in Iraq) did not allow the field researchers to go to all the villages that we had planned to visit in the Nineveh Plain, since their safety could not be guaranteed given that a number of ISIS affiliates may still be hiding in this area close to Mosul.

The wild plant species mentioned by the informants were collected, when available, and identified by the authors according to the *Flora of Turkey and the East Aegean Islands* [22]; this resource was chosen because the *Flora of Iraq* is still unfinished (with only five completed volumes [23]). The collected specimens were stored at the Herbarium of the Department of Environmental Sciences, Informatics, and Statistics, Ca’ Foscari University of Venice (UVV, specimens KURD66-KURD106) and at the Herbarium of the Estonian University of Life Sciences (TAA, specimens KURD01-KURD59). When specimens were not available, a possible identification was attempted by asking the informants to describe the plant and its habitat as well as possibly show mobile phone pictures, and by comparing the recorded folk name with the most exhaustive dictionary of Kurdish plant folk names [24].

Nomenclature follows the standards set by The Plant List database [25], while plant family assignments follow the current Angiosperm Phylogeny Group designations [26]. Local plant names were reported in the Latin alphabet; among the phonemes that do not occur in English, it is worth mentioning that the voiceless velar fricative was reported as “x” and voiceless uvular stop as “q”.

### Data analysis

Collected data were compared with the entire Kurdish wild food ethnobotanical literature available in English or Russian [6–13, 17, 27, 28] and with that of various territories in the Near/Middle East and the Caucasus, where ethnobotanical field studies focusing on wild food plants have been sporadically conducted during the past decades [29–37].

Finally, the most comprehensive reviews on the wild food and medicinal plants used in Iraq [38] and the entire Near East/Caucasus [39] were considered.

## Results and discussion

### South Kurdish foraging

Table 1 presents the wild food plants reported by the informants as gathered and consumed. In the table, along with the botanical taxa, families, and voucher codes, we report the folk names that we recorded in the study area, as well as the used plant parts, their traditional culinary uses, and the quotation frequency for both religious groups (proportion of the overall informants citing the food use of a given folk taxon).

**Table 1:** Traditionally gathered wild food plants recorded among Muslim and Kakai Kurds in the study area

Botanical taxon/taxa, family, and voucher specimen code(s)	Recorded local name(s)	Used parts	(Etic) taste characteristics	Traditional culinary use	Quotation
*Allium ampeloprasum* L., KURD11	Koraya, Qorada, Qurada	Aerial parts	Pungent	Seasoning	M
*Allium iranicum* (Wendelbo) Wendelbo, Amaryllidaceae^#^	Keniwal	Whole plant	Pungent	Bread seasoning; snack	K, M
*Allium koelzii* (Wendelbo) Perss. and Wendelbo, Amaryllidaceae, KURD20	Kol	Young leaves	Light pungent and sweetish	Cooked	M
*Allium paradoxum* (M. Bieb.) G. Don, Amaryllidaceae^#^	Karat^K^, Knal^K^, Piçek^K^, Siçek^K^	Leaves	Light pungent and sweetish	Consumed raw as *sawza*; bread and yogurt seasoning; cooked with rice or bulgur; preserved in brine (lacto-fermented)	KK, M
*Anchusa azurea* Mill., Boraginaceae, KURD100	Gezerwan, Gozerwan, Gurmiza	Young aerial parts	Herbaceous	Boiled	K, MM
*Arum rupicola* Boiss., Araceae, KURD92, possible other *Arum* spp., and *Dracunculus vulgaris* Schott, Araceae, KURD99	Kardi, Kardu, Xas	Leaves	Pungent	Boiled in “sumac water” (suspension of water and sumac fruits) and then cooked in various ways (often with rice/bulgur and wild leek); preserved in brine (lacto-fermented) or dried (sometimes in necklaces)	KK, MMM
*Asparagus* sp., Asparagaceae^#^	Marije^K^, Marijok^K^	Shoots	Slightly bitter	Cooked with rice	K
*Bongardia chrysogonum* (L.) Spach, Berberidaceae, KURD96	Gabla, Galba	Shoots	Herbaceous (earthy)	Cooked	M
*Bunium paucifolium* DC., Apiaceae, KURD89	Dobeldobana*, Dobel*	Tubers	Sweetish and crunchy	Snack; boiled	KK
*Chaerophyllum bulbosum* L., Apiaceae^#^	Zarkazawi, Zargazewi	Tubers	Sweetish and crunchy	Snack	K, M
*Citrullus colocynthis* (L.) Schrad., Cucurbitaceae^#^	Jijalek*, Gujalek*, Gumpshila^K^	Unripe fruits	Bitter	Snack (as a medicinal food for treating kidney dysfunctions)	K, M
*Crataegus* spp., Rosaceae^#^	Gewask^K^, Gwaiş	Fruits	Slightly astringent and sweet	Snack	K, M
*Crocus biflorus* Mill.^#^ and possibly other *Crocus* spp., Iridaceae	Pifok, Piçek, Pijok^K^, Pişok, Pivok	Corms (after removal of fibrous tunic)	Herbaceous (earthy) and crunchy	“Social snack”	KK, MM
*Erodium cicutarium* (L.) L'Hér. and *Erodium moschatum* (L.) L'Hér., Geraniaceae, KURD79, KURD77	Agilaklak*, Darzila, Dendulaklak*, Giaderzile, Menkarlaklak*	Young infructescences	Herbaceous	Snack (sometimes considered a medicinal food for treating stomach-aches)	KK, MM
*Foeniculum vulgare* L., Apiaceae KURD88	Hazola, Rasiana*	Young leaves	Aromatic	Raw as *sawza*	K, M
*Geranium tuberosum* L., Geraniaceae^#^	Pushien	Tubers	Crunchy	Snack; preserved in brine (lacto-fermented)	M
*Glycyrrhiza glabra* L.^#^ Fabaceae	Balek*	Young stems (peeled)	Sweet	Snack	K
*Gundelia turnefortii* L., Asteraceae, KURD97	Çinger^K^, Kinger	Internal parts of the tender whorls and upper part of the root; seeds (*sesi*)	Slightly bitter (whorls); nutty (seeds)	Whorls: boiled; preserved in brine (lacto-fermented); seeds: boiled in salty water, then roasted, and consumed as a “social snack”	KKK, MMM (whorls); K, M (seeds)
*Imperata cylindrica* (L.) Raeusch., Poaceae, KURD95	Piazoka	Young aerial parts	Herbaceous	Raw as *sawza*	M
*Johrenia aromatica* Rech. f., Apiaceae, KURD69	Baraza	Aerial parts	Aromatic	Recreation tea; “social snack” (this is consumption sometimes considered as a food medicine for treating kidney disease)	M
*Lathyrus* sp., Fabaceae^#^	Polka	Young fruits	Herbaceous	Snack	M
*Malus orientalis* Uglitzk. ex Juz., Rosaceae^#^	Sevelok, Sevun, Siev^K^	Unripe fruits, fruits	Astringent and sour (unripe fruits); sour and sweet (ripe fruits)	Snack	KK, M
*Malva neglecta* Waller, Malvaceae, KURD03	Talaka^K^, Tolaga^K^, Tolka, Tollaka^K^, Xobas*	Leaves, stems (peeled), and fruits	Herbaceous and mucilaginous	Leaves: cooked with eggs, *sarma*; soups, preserved in brine (lacto-fermented); sometimes considered a medicinal food for treating heart disease; stems and fruits: snacks	KKK, MMM
*Matricaria chamomilla* L., Asteraceae, KURD 84	Beibun, Gulaçarma^K^, Gulaçarmala, Gurlinka	Flowering tops	Aromatic	Recreational tea	KK, M
*Mentha longifolia* (L.) Hudson and *Mentha spicata* L. Lamiaceae, KURD73, KURD08	Ping, Punga	Leaves	Aromatic	Seasoning (esp. yogurt); recreational tea (often with raisins)	KK, MMM
*Myrtus communis* L., Myrtaceae, KURD103	Mert	Leaves	Aromatic	Recreational tea	M
*Nasturtium officinale* R.Br., Brassicaceae, KURD57	Çuzala, Kuzala, Pandirpoza^K^, Pizala^K^, Xuzala	Aerial parts	Pungent	Raw as *sawza*	KK, MM
*Ornithogalum balansae* Boiss. and possibly other *O*. spp., Asparagaceae, KURD94	Aerial parts: Gelik, Glexa^K^ Bulbs: Formaşişana, Hormçiçek, Hurmaşişana, Hurmatsitsana, Şimişak^K^	Aerial parts and bulbs	Herbaceous (aerial parts). bitter and crunchy (bulbs)	Aerial parts: cooked; bulbs: snack, cooked	KK, MM
*Petasites albus* (L.) Gaertn., Asteraceae^#^	Kaşma	Leaves	Slightly bitter	Cooked	M
*Pistacia* *atlantica* Desf., Anacardiaceae, KURD102	Kaskauan^K^, Kaskavaniş, Kaşakau^K^, Kaskuan	Unripe fruits	Resinous	Seasoning *mastaw* (ayran) and *terhana* (mixture of bulgur/grains and yogurt); preserved in brine and consumed as a side-dish^K^	KK, MMM
*Pleurotus* and possibly *Agaricus* spp., Pleurotaceae^#^	Karçik^K^, Karg, Karzik, Xarçek, Xarzek, Xuarek	Fruiting bodies	Mushroom-like	Boiled and then fried	KK, MMM
*Portulaca oleracea* L., Portulaceae, KURD27	Barpina, Palapina, Parpina	Aerial parts	Herbaceous (mineral) and crunchy	Raw or cooked	K, MM
*Prosopis farcta* (Banks and Sol.) J.F.Macbr., Fabaceae*#*	Xarnik*, Xaşxaşa*	Seeds	Sweetish and nutty	Snack (sometimes consumed as a medicinal food for treating stomach-ache and diarrhoea in children)	K
*Prunus* *arabica* (Olivier) Meikle^#^ Rosaceae	Bayaf, Bayu, Baui*, Nabyk^K^	Kernels	Very bitter	Boiled with salt and then consumed as a snack (preserved in the same brine)	K,M
*Prunus cerasifera* Ehrh. and *Prunus microcarpa* C. A. Mey., Rosaceae^#^	Gelas*, Halu, Zardalu	Unripe fruits	Sour and astringent	Snack	K, M
*Prunus* *webbii* (Spach), Vierh*.*, Rosaceae^#^	Çakalove*	Unripe fruits	Sour and astringent	Snack	K
*Quercus infectoria* G. Olivier, Fagaceae, KURD07	Şokabaru	Honeydew (”Kurdish manna”): *Gazo:* collected on oak leaves. *Şoka:* collected on unripe acorns, (Fig. 9)	Sweet	Syrup	M
*Quercus petraea* (Matt.) Liebl. and possibly *Quercus* brantii Lindl.^#^, Fagaceae, KURD101	Baru, Şabalu	Unripe and ripe fruits	Astringent	Unripe fruits: snack; ripe fruits: roasted or boiled and then roasted; eaten with honey against stomach-ache; preserved dried (mainly in the past)	KK, MM
*Rheum ribes* L., Polygonaceae, KURD104	Rewas	Leaf petioles	Sour	“Social snack”	K, MM
*Rubus ulmifolius* Schott, Rosaceae^#^	Alga*, Tuturk	Fruits	Sweet	Snack	K, M
*Rumex acetosa* L., *Rumex crispus* L., and possibly other *Rumex* spp., Polygonaceae, KURD50, KURD81	Trşoka, Truska, Turşka^K^, Xamga*	Leaves	Sour	Raw as *sawza* (*R. acetosa*); *sarma* (*R. crispus*); tea for treating stomach-ache	KK, MM
*Satureja thymbra* L., Lamiaceae, KURD04	Hasola, Iatra, Jatra, Zatra	Aerial parts	Aromatic	Seasoning	MMM
*Scorzonera papposa* DC., Asteraceae^#^	Damkoz^K^, Gazer^K^, Halaluk, Hapaluk, Haplog, Karkoza, Pesbala	Roots and leaves	Sweetish (roots); herbaceous (leaves)	Roots: raw snack (sometimes considered a medicinal food for treating stomach-ache); preserved in brine (lacto-fermented); leaves: cooked in yogurt	KK, MMM
*Silybum marianum* (L.) Gaertn. and (more rarely) *Carduus pycnocephalus* L., Asteraceae, KURD85, KURD22	Çaubaza, Kalagan, Kalaguana^K^, Kalangana, Kalgana, Kalxana^K^, Kerbaşa, Kevar, Kosep*, Xalxana^K^	Young stems (peeled)	Slightly bitter and crunchy	“Social snack”	K, MMM
*Sinapis arvensis* L. and (more rarely) *Raphanus raphanistrum* L., Brassicaceae, KURD71, KURD75	Fijiela*, Gulasarda^K^, Tavar, Teveroka, Torpoka^K^, Turuoka, Xartala, Xatala^K^	Young stems (peeled) and leaves	Slightly pungent	Stems: snack; leaves: soup	K, MM
*Smyrnium cordifolium* Boiss., Apiaceae^#^	Gnor, Narima	Stems (peeled)	Aromatic	Snack	MM
*Solanum nigrum* L., Solanaceae^#^	Arrosalà^K^	Fruits	Herbaceous	Snack	K
*Terfezia* and *Tirmania* spp., Terfeziaceae, KURD106	Dolaman^K^, Dombalan, Dumaran^K^	Fruiting bodies	Mushroom-like	Boiled and then cooked, often with eggs and onions; roasted; preserved in brine (lacto-fermented); tea for treating eye inflammations	KKK, MMM
*Thymus* sp., Lamiaceae^#^	Asbiela	Aerial parts	Aromatic	Seasoning	M
*Tordylium aegyptiacum* (L.) Lam., Apiaceae, KURD82	Gurame*, Gurgemi, Nanafalla	Seeds	Aromatic	Snack; seasoning	K, MM
*Tragopogon collinus* DC., Asteraceae^#^	Şing	Leaves and roots	Leaves: herbaceous, roots: sweetish	Cooked	M
*Tribulus terrestris* L., Zygophyllaceae^#^	Peikola^K^	Unripe fruits and seeds	Herbaceous (pea-like)	Unripe fruits: snack; Seeds: boiled	KK, M
*Tulipa* *montana* Lindl.^#^ and possibly other *Tulipa* spp., Liliaceae	Melaqa	Bulbs	Sweetish and crunchy	Snack	MM
*Vicia ervilia* (L.) Willd., Fabaceae, KURD87	Gadana^K^	Young fruits	Herbaceous (pea-like)	Snack	KK
*Ziziphus jujuba* Mill. Rhamnaceae, KURD91	Knar	Fruits	Sweetish and sour	Snack	M
Unidentified (Amaryllidaceae?) sp.	Zaxari^K^, Zotka^K^, Zuotka^K^	Underground parts		Snack (sometimes considered a medicinal food for treating heart diseases)	KK
Unidentified (Apiaceae?) sp.	Şawbo^K^	Fruits		Seasoning	K
Unidentified sp.	Danteşkara^K^	Fruits		Snack; cooked	K
Unidentified sp.	Fetr*	Underground parts		Snack	M
Unidentified sp.	Damkos^K^	Underground parts		Snack	K

*KKK* very commonly quoted by Kakai Kurds (more than 40% of informants), *KK* commonly quoted by Kakai Kurds (10–40% of informants), *K* rarely quoted by Kakai Kurds (less than 10% of informants), *MMM* very commonly quoted by Sunni Muslim Kurds (more than 40% of informants), *MM* commonly quoted by Sunni Muslim Kurds (10–40% of informants), *M* rarely quoted by Sunni Muslim Kurds (less than 10% of informants)

^#^Identification made on the basis of plant description, folk names, and/or pictures provided by the informants

*Local name recorded in the multi-ethnic (Kurdish, Arab, Shabak) village of Sherkan

^K^Local name recorded in Kakai villages only

In total, 54 folk wild plant taxa and 2 folk wild fungal taxa (corresponding to 65 identified botanical taxa and 4 fungal taxa) were recorded, while 5 plant folk taxa remained unidentified. This remarkable number of currently gathered wild food plants shows that Kurdistan still represents an extraordinary hotspot of traditional foraging in the world. This is confirmed also by all recent field studies conducted in different areas inhabited by Kurds [6–9, 11–13, 17], especially if we compare them with research outcomes recently found in surrounding non-Kurdish areas [34–36].

Most of the quoted wild food plants are wild vegetables, and the majority of them are mainly consumed raw, both as wild greens and especially as snacks. The former refer to the Kurdish tradition of consuming raw herbs as a side-dish (*sawza*): both wild and cultivated greens (i.e. watercress, coriander, parsley, dill) appear on the table and are picked up by hand, before using the flatbread to scoop up the main dish. The latter (snacks) represent plant parts which are gathered and consumed raw on the spot.

**Fig. 4:**
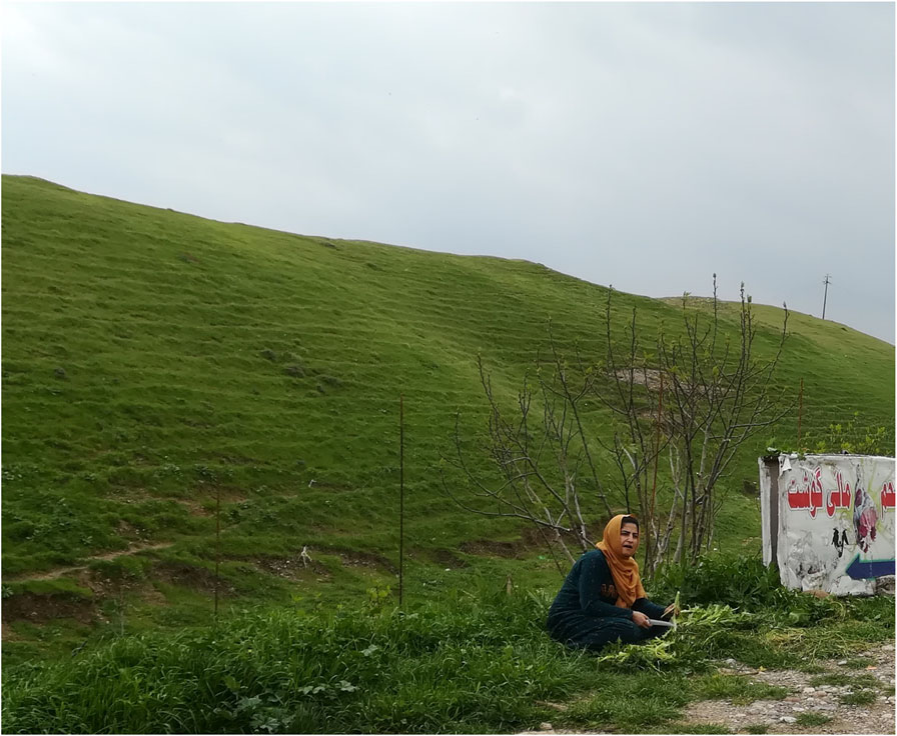
Kurdish woman peeling stalks of *Silybum marianum*

**Fig. 5:**
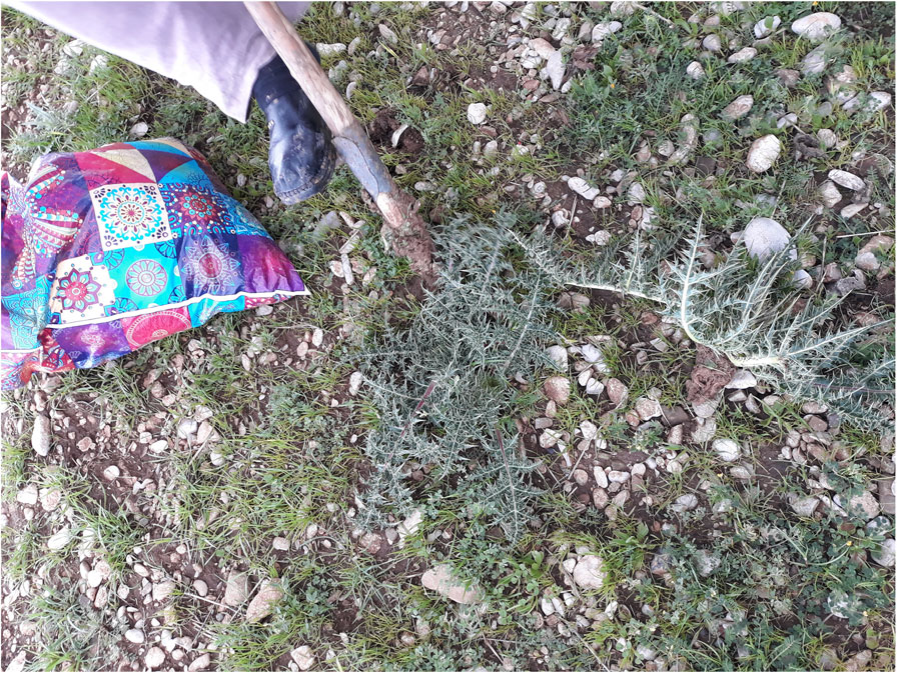
Kurdish woman collecting *Gundelia turnefortii* by digging out the young aerial parts and the upper part of the root using a hoe

Normally, thorny plants are gathered using a big knife for removing the thorny parts (Fig. 4), while a few others (i.e. *Gundelia turnefortii*) are dug out using a hoe (Fig. 5 and [40]) and gloves and taken home where they are further processed to eliminate the thorny outer parts.

Some wild plant ingredients, however, are cooked, while very few recorded wild plants are used as seasoning. Often, some green plant parts are preserved in brine via lacto-fermentation (especially in the past) or more often nowadays simply frozen.

### The importance of snacks

**Fig. 6.**
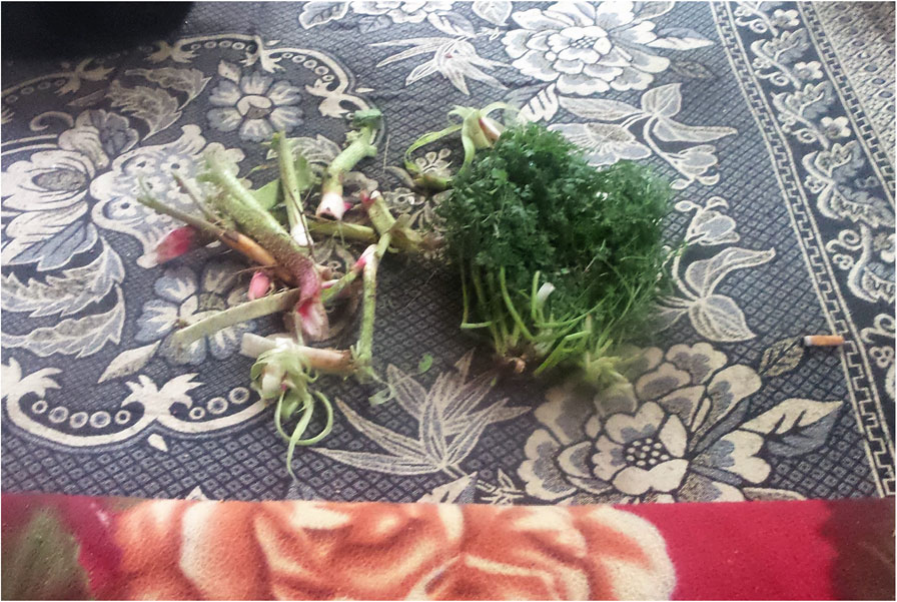
Raw *Rheum ribes* stalks and raw aerial parts of *Johrenia aromatic* ready for consumption as domestic “social snacks”

More than 60% (36 out of 59) of the overall recorded folk plant taxa (54 identified folk taxa and 5 unidentified folk taxa) are consumed as snacks, i.e. raw on the spot. Several of these snacked plants seem to be predominately gathered by men and especially by male teenagers while they are out in nature. Some of them do enter into the domestic arena and they represent “social snacks”, i.e. they are consumed raw during family and social gatherings (Fig. 6).

Women forage instead mainly wild vegetables (especially weeds) that are normally further processed and cooked, or even simply consumed raw as a side-dish (*sawza*).

Raw plant snacks are indeed an interesting phenomenon in food anthropology: they have rarely been reported in ethnobotanical studies [41], as they are probably not systematically captured in the interviews, which are not normally designed to address this hidden subject. According to the literature we analysed, in Middle Eastern ethnobotanical studies, they have been sometimes counted together with plants that are consumed raw within the domestic arena, as side-dishes or salads.

Although these limitations make a robust comparison with other ethnobotanical studies very difficult, we formulate here the hypothesis that snacks may have emerged during the development of mobile pastoralism. Personal observations and other ongoing field studies conducted within our research group confirm that in areas where pastoralism has been predominant in the past centuries (i.e. Sardinia), edible thorny Asteraceae, which were traditionally peeled and consumed on the spot while bringing herds to pasture, play a crucial role in the local folk diet. It is worth mentioning here that foraging pastoralist practices were possibly the origin of two currently very popular vegetables in the Mediterranean and the Near East: artichoke, which was domesticated in the central Mediterranean area a couple of thousand years ago [42], and wild *akoob/kenger* (*Gundelia turnefortii*), still widely used in the Arabic, Israeli, and Kurdish cuisine [13, 39, 43].

Among the most uncommon wild plant snacks we recorded, it is important to highlight the variety of underground food items, such as those of *Allium*, *Bunium*, *Chaerophyllum*, *Crocus*, *Geranium*, and *Tulipa* spp.

All the recorded plant snacks present a particular sensory characteristic (Table 1): they are crunchy. This observation suggests the possible role of plant texture and especially crunchiness—maybe even more than their taste—in shaping cultural preferences for specific vegetable ingredients in predominately pastoralist cultures.

**Fig. 7:**
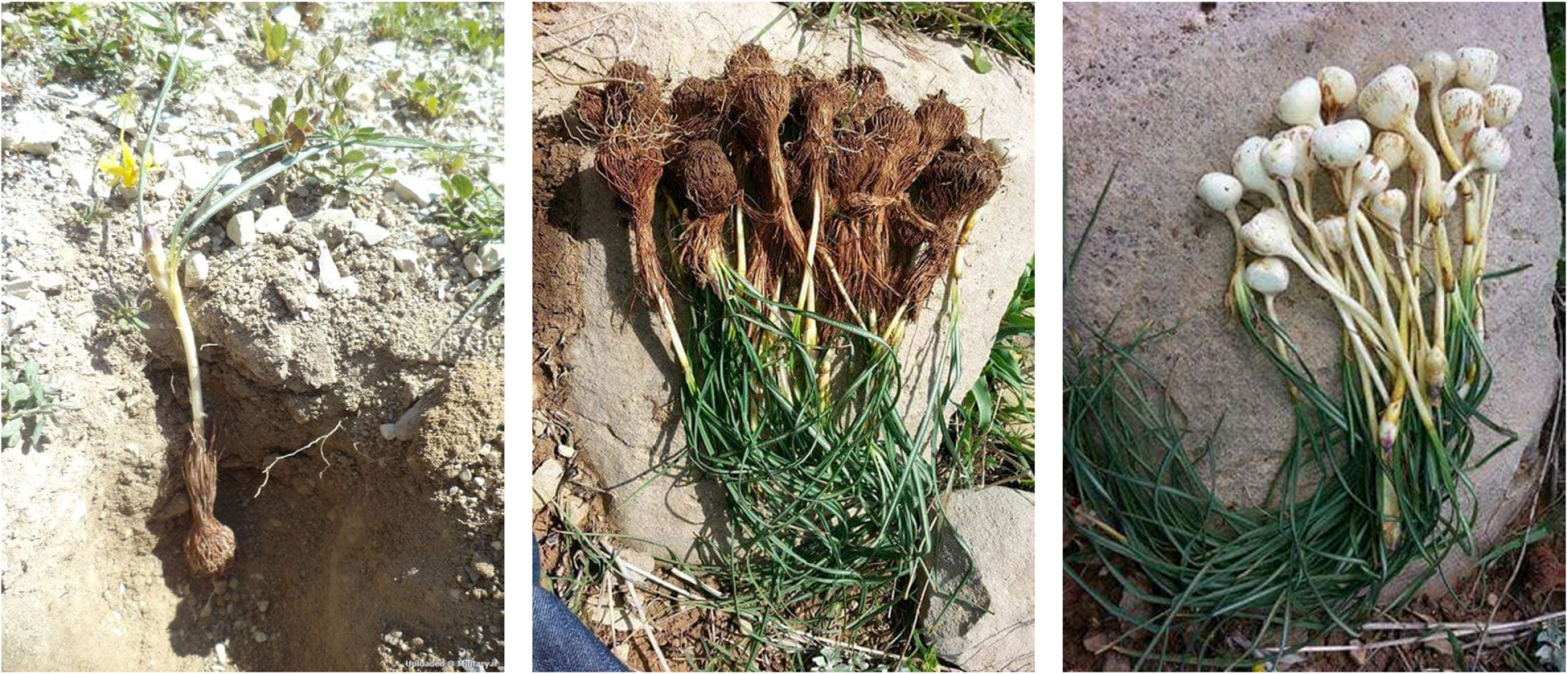
Extraction of *Crocus* corms before and after removal of the fibrous tunic (photo courtesy of Xalid Rasul)

Among the recorded plant snacks, it is interesting to underline the current consumption of wild crocus corms and tulip bulbs. The gathering of raw crocus corms (Fig. 7) in our study area is still very commonly practiced, even by young people, and it is popular also in some areas of Turkish Kurdistan and of Jordan [11, 12, 31].

Bulbs of wild mountain tulips (*Tulipa montana*) were instead reported to be consumed in Iraqi Kurdistan in the past century [38] when they were even said to be sold at the Mosul market, as well as gathered in Beluchistan and Afghanistan [39, 44], while *Tulipa armena* bulbs have recently been reported to be (very rarely) consumed raw in Turkish Kurdistan [45]. Consumption of cooked tulip bulbs has been known to occur in Europe during times of famine, such as the last century in the Netherlands [46], where interest in tulip domestication and the celebration of the beauty of its flowers, which possibly started in Persia during the tenth century, arrived in the sixteenth century from the Ottoman Court [47]. To our surprise, crocuses and tulips are not highly regarded in our study area as ornamentals in home gardens, where *Fritillaria imperialis* instead represents the most desired bulbous ornamental plant.

### Muslim Kurd vs. Kakai Kurd wild food ethnobotany

**Fig. 8:**
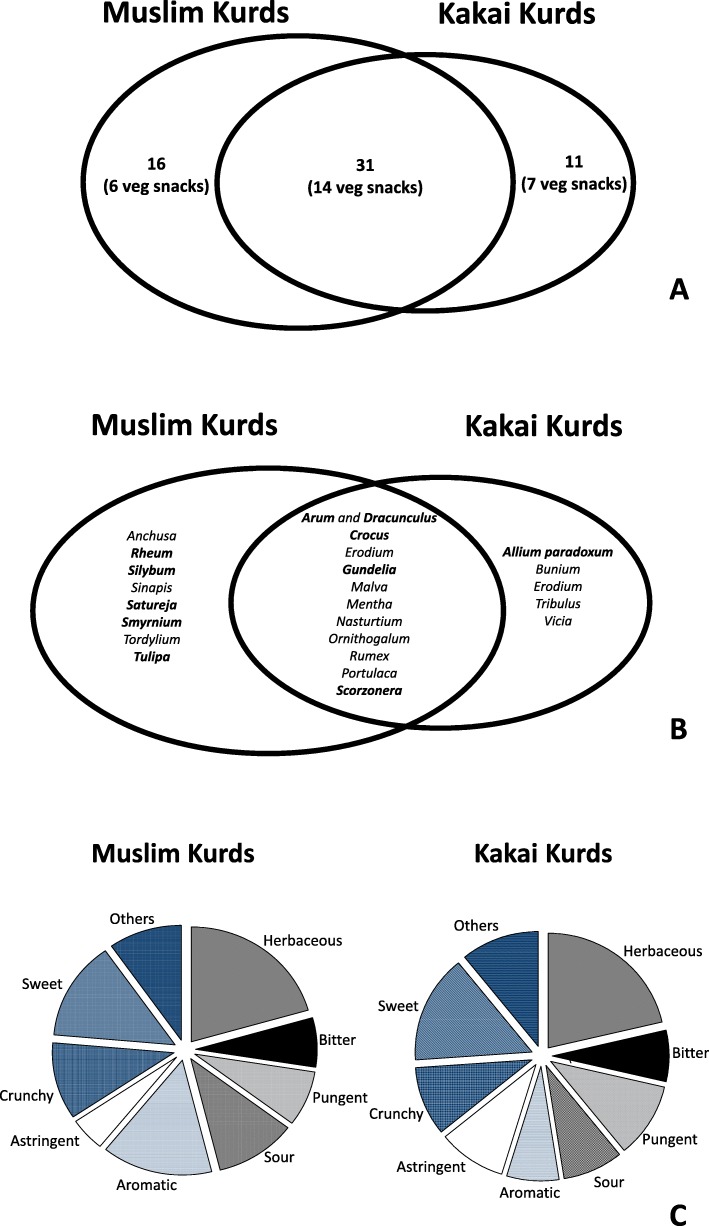
**a** Comparison of the overall wild food plant taxa and vegetable snacks quoted by Muslim and Kakai Kurds. **b** Comparison of the most commonly quoted wild vegetable genera/species reported by Muslim and Kakai Kurds (in bold we indicated *non-weedy* genera/species). **c** Comparison of the predominant tastes of the wild plants quoted by Muslim and Kakai Kurds (data also take in account their quotation frequency, see Table 1)

Figure 8 illustrates the overlap between wild food ethnobotanies of Muslims and Kakais.

Figure 8a reports the overall gathered folk plant taxa and, in brackets, the vegetable snacks, while Fig 8b illustrates the *most commonly quoted* wild vegetable genera or species. Taxa that are not weeds (defined here as plants that “grow entirely or predominantly in situations markedly disturbed by man without being deliberately cultivated” [48]) are reported in bold.

Figure 8c shows instead the predominance of the wild food plant tastes.

No remarkable differences could be found among the two communities in terms of overall gathered plant taxa and their wild vegetable snacks (Fig. 8a). However, the wild food ethnobotany of the Kakai seems to be more restricted, and commonly quoted non-weedy wild vegetables are prevalent among Muslims, thus possibly disclosing stronger traces of mobile pastoralism within this latter group (Fig. 8b). This is confirmed by the comparison of the predominance of the wild food plant tastes (Fig. 8c), where Muslim Kurds seem to slightly prefer aromatic and crunchy taste/texture, which are common features of non-synanthropic Apiaceae vegetable snacks that are normally gathered in the mountains.

The available ethnographic literature may confirm this finding, since the traditional subsistence economy of Yarsanis/Kakais, which emerged in Iran during medieval times, was mainly based upon small-scale horticulturalism and handicrafts (weaving) [49], whereas most Kurds were more frequently nomadic and semi-nomadic pastoralists [50].

In other studies that we have conducted in recent years in the Balkans and the Middle East, we have demonstrated that religious groups living in the same environment may sometimes show different ethnobotanies, possibly because endogamic patterns play an important role in influencing vertical and, to less extent, also oblique transmission of folk plant knowledge [13, 51–54].

In the current study, however, we did not observe remarkable differences between the two studied communities. One possible reason can perhaps be found in the large utilization of wild plant snacks: we observed that these are mainly gathered in the study area by young male community members, and therefore, horizontal (peer-to-peer) transmission of plant knowledge may have been predominant in our sample.

### Comparison with the Middle Eastern ethnobotany and human ecology of Kurdish foraging

**Fig. 9:**
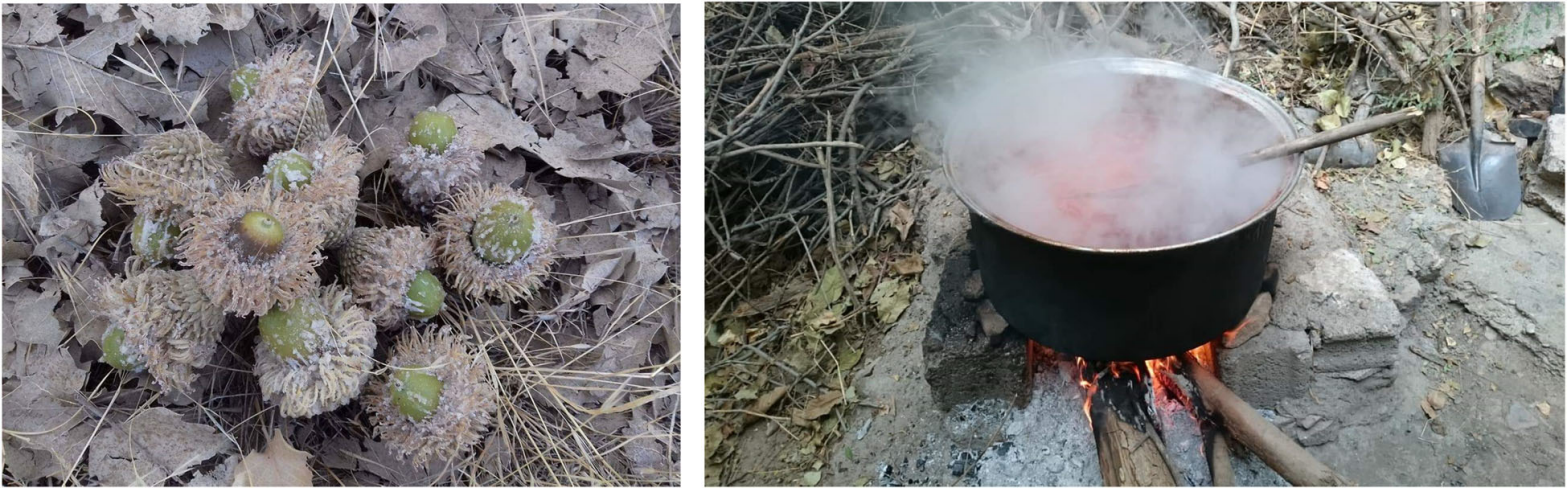
Kurdish manna on acorns and extracted in hot water (photo courtesy of Hamza Zahir)

Comparison of the current data with some of the most recent wild food ethnobotanical literature on the Middle East shows that a few wild plants have only rarely been recorded as being consumed: this is the case for the botanical genera *Tulipa*, *Lilium*, *Crocus*, *Dracunculus*, *Johrenia*, and *Bongardia*. Among the most uncommon customs linked to plants, we recorded the tradition of gathering the famous “Kurdish manna” (Fig. 9), well known in historical accounts of travels to Kurdistan [55], and references therein], produced by a few oak acorn species, and whose consumption in syrup is still very highly esteemed by elderly Kurds. Oak dew is known to be produced only under certain climatic conditions during a few days in early June (on the leaves) and in September (on the fruits) and not every year. The acorns or leaves covered by the dew are boiled in water and filtered, and the resulting solution is evaporated (Fig. 9).

Moreover, we analysed the data in terms of possible human ecological origin, i.e. calculating the proportion of weeds among the quoted wild vegetables as a proxy for analysing possible horticultural/sedentism-driven foraging patterns.

Table 2 compares the percentage of weedy vegetables in the current study area and in a few selected wild food ethnobotanical studies conducted among other surrounding populations of the Middle East and the Mediterranean, and the predominance of non-weeds among Kurds and Azeris is remarkable when compared with that recorded among Arabs and especially ethnic Assyrians and Greeks.

**Table 2:** Proportion of weedy plants quoted in the current study and other ethnobotanical field research recently conducted in surrounding regions

Ethnic group, area [bibliographic reference]	Percentage of weeds in the total gathered wild vegetables
Sorani Kurds, Southern Iraqi Kurdistan, present study	33
Zaza Kurds, Eastern Turkey [6]	38
Azeris, Azerbaijan [51]	42
Yezidi diaspora, Armenia [9]	43
Kurmanji Kurds, Eastern Turkey [12]	52
Arabs, Lebanon [34]	54
Assyrians, Northern Iraqi Kurdistan [13]	69
Griko (Greek) diaspora, SE Italy [56]	70

This data suggests the idea that Kurds may have shaped their foraging habits upon their nomadic pastoralist subsistence economy, while the sedentism and horticulturalism of the Fertile Crescent have left heavy traces in the foraging patterns of Assyrians [13] and later—when the post-Neolithic foodscape moved westwards—Greeks and other Mediterraneans.

## Conclusion

The overall gathered data not only show a remarkable resilience of foraging traditions in Southern Kurdistan, but also document the food consumption of several wild plant ingredients as raw snacks, i.e. crocus corms and tulip bulbs. While no very significant divergences were found among the two studied religious communities of Muslim and Kaki Kurds, among the Muslim Kurds, non-weedy plants were clearly more prevalent among the most commonly quoted wild vegetables, as Kakai Kurds have historically been more horticulture oriented. At the same time, the large prevalence of snacks, especially among Muslim Kurds, confirms robust traces of pastoralism in the Kurdish foraging of wild foods.

Our study calls for further field surveys in surrounding regions of the Middle East, Caucasus, and Eastern Mediterranean aimed at analysing how TEK concerning wild plants change across time and space and for a better understanding of the diachronic trajectories of the use of wild plant foods before and after the development of agriculture until today.

Finally, the recorded Kurdish bio-cultural food heritage could find concrete applications in rural development projects aimed at promoting small-scale food products and eco-tourism, considering the very difficult times this area has gone through and is still partially going through.

## Data Availability

The main data are incorporated into the research article.
